# Association of Differentiation State of CD4+ T Cells and Disease Progression in HIV-1 Perinatally Infected Children

**DOI:** 10.1371/journal.pone.0029154

**Published:** 2012-01-11

**Authors:** Elizabeth R. Sharp, Christian B. Willberg, Peter J. Kuebler, Jacob Abadi, Glenn J. Fennelly, Joanna Dobroszycki, Andrew A. Wiznia, Michael G. Rosenberg, Douglas F. Nixon

**Affiliations:** 1 Division of Experimental Medicine, Department of Medicine, University of California San Francisco, San Francisco, California, United States of America; 2 Jacobi Medical Center, Bronx, New York, United States of America; University of Cape Town, South Africa

## Abstract

**Background:**

In the USA, most HIV-1 infected children are on antiretroviral drug regimens, with many individuals surviving through adolescence and into adulthood. The course of HIV-1 infection in these children is variable, and understudied.

**Methodology/Principal Findings:**

We determined whether qualitative differences in immune cell subsets could explain a slower disease course in long term survivors with no evidence of immune suppression (LTS-NS; CD4%≥25%) compared to those with severe immune suppression (LTS-SS; CD4%≤15%). Subjects in the LTS-NS group had significantly higher frequencies of naïve (CCR7+CD45RA+) and central memory (CCR7+CD45RA−) CD4+ T cells compared to LTS-SS subjects (p = 0.0005 and <0.0001, respectively). Subjects in the rapid progressing group had significantly higher levels of CD4+ T_EMRA_ (CCR7−CD45RA+) cells compared to slow progressing subjects (p<0.0001).

**Conclusions/Significance:**

Rapid disease progression in vertical infection is associated with significantly higher levels of CD4+ T_EMRA_ (CCR7−CD45RA+) cells.

## Introduction

Various studies have sought to determine an association between the control of HIV-1 viremia and magnitude of the HIV-1 specific immune response [1], [2], [3], [4], [5]. The results have been inconsistent. The qualitative characteristics of the HIV-1-specific T cell response have become the focus of intense study and it has been suggested that the inability of these responses to control viremia is due to a failure of these cells to fully differentiate [6].

In contrast to other chronic viral infections such as CMV, HIV-1 infection appears to result in a maturational block in the generation of the HIV-1-specific T cell responses with skewing toward an effector memory, T_EM_, phenotype [6]. This seems to result in an overall decrease in the frequency of fully differentiated effector memory, T_EMRA_, cells [6], [7]. We have previously shown that the frequency and absolute numbers of CD8+ HIV-specific T_EMRA_ cells in early HIV-1 infection negatively correlate with the future viral load set point [8]. As CD4+ T cells are also known to be important in the control of HIV-1 viremia[9], [10], [11], [12], [13], [14], [15], [16], [17], [18], we sought to determine whether alterations in CD4+ T cell subpopulations were associated with disease progression. We chose to study a population of vertically infected children, and categorized them into two progression groups based on CD4% values using revised guidelines published by the CDC in 1994 [19], subjects with no immune suppression (LTS-NS; CD4%≥25%), and subjects with severe immune suppression (LTS-SS; CD4%≥15%). Surprisingly, we found striking differences in the differentiation phenotype of CD4+ T cells between the two groups.

## Results

### Subject Cohort Characteristics

We analyzed peripheral blood samples from 58 children and adolescents with vertically acquired HIV-1. As described in Materials and Methods, these subjects were divided into two groups of immunological progression based on CDC guidelines. The characteristics of both groups are described in Table 1.

**Table 1:** Patient cohort characteristics.

Immunological Category	N	CD4%	LVL^a^	Age (y)	Sex	Race^b^
No immune suppression - LTS-NS (CD4% ≥25)	30	29.8% (27.1; 34.8)	3.65 (2.85; 4.14)	13.8 (10.9; 16.6)	M = 15 F = 15	H = 9 AA = 20
No immune suppression - LTS-NS (CD4%<15)	28	8.25% (5.5; 11.5)	4.79 (4.37; 5.11)	15.6 (12.5; 18.1)	M = 11 F = 17	H = 13 AA = 13
TOTAL	58	24.5% (8.25; 31.0)	4.28 (3.61; 4.81)	14.3 (11.6; 17.52)	M = 26 F = 32	H = 22 AA = 33

a Log viral load.

b H = Hispanic; AA = African American.

As the children were categorized according to percentage CD4+ T cell count, it was not surprising to find a statistically significant difference in the viral loads between the two groups. Of particular note, all of the patients had some level of ongoing viral replication, as none of them maintained consistently undetectable viral loads. The LTS-NS group contained more African-Americans than the LTS-SS group, but this did not reach significance. The LTS-SS group was slightly older than the LTS-NS group, but again this did not reach significance. There were no significant differences in treatment regimen or adherence levels between the two clinical groups.

### Comparison of Differentiation Profiles of Bulk and HIV-1-specific CD8+ T cells Between Progression Groups

We first characterized the HIV-1-specific CD8+ T cell population in the two groups. We hypothesized that there would be more fully differentiated CD8+ T_EMRA_ cells in the LTS-NS subjects compared to LTS-SS subjects, both in the total CD8+ T cell population and in Gag-specific CD8+ T cells, as has been observed from studies from adult HIV-1 infected cohorts [20], [21]. We performed surface staining and intracellular cytokine staining on 17 LTS-NS subjects and 15 LTS-SS subjects, stimulating PBMC with single Gag peptides.

**Figure 1:**
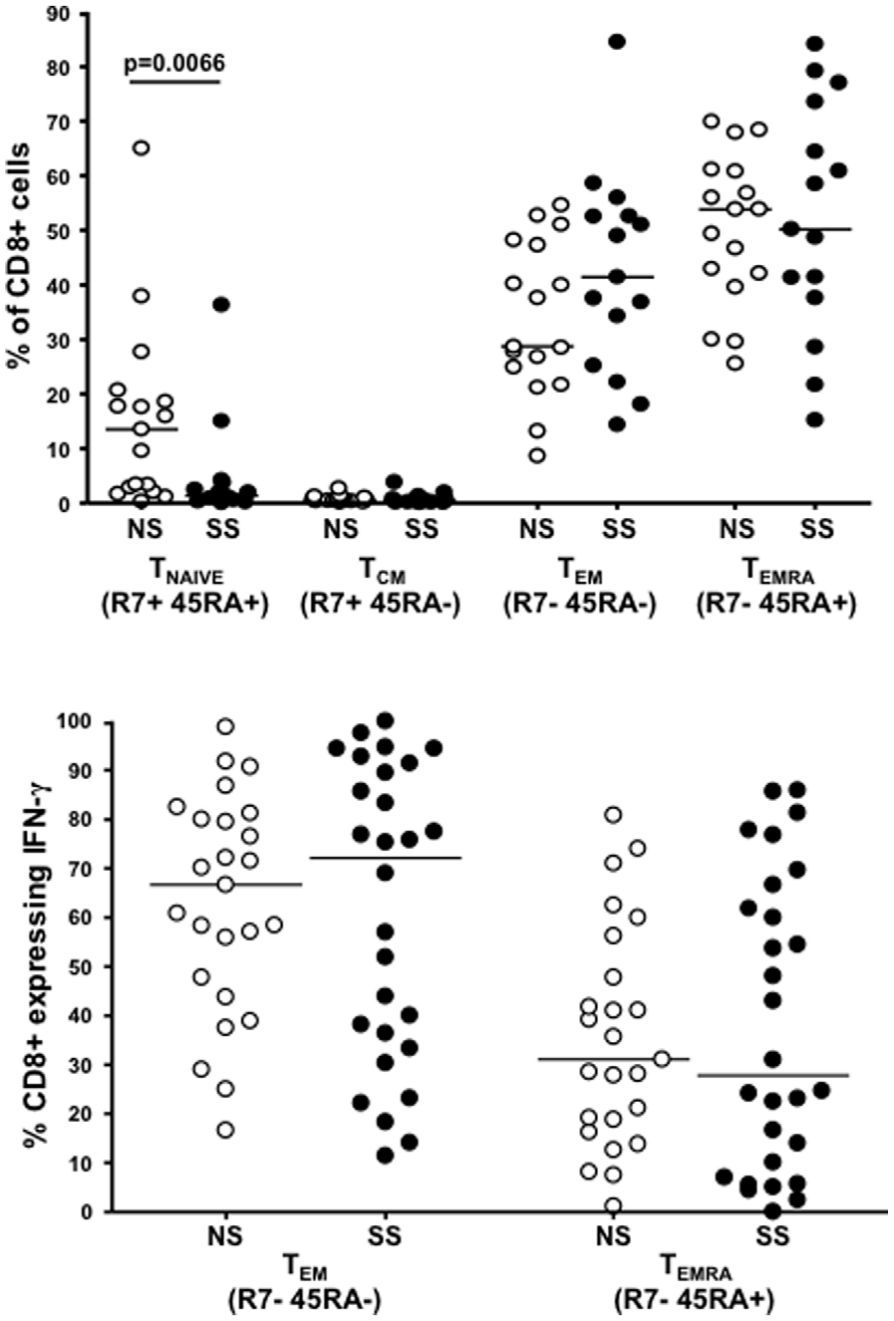
Comparison of differentiation profiles of total CD8+ T cells between progression groups (A) and Comparison of differentiation profiles of Gag-specific effector CD8+ T cells between progression groups (B). We used expression of CCR7 and CD45RA to categorize CD8+ T cells into one of four differentiation phenotypes. Filled circles (•) represent LTS-SS patients and empty circles (○) represent LTS-NS patients. The line in each column represents the median and the differentiation phenotype is beneath each column.

Surface staining of the total CD8+ T cell population revealed a significantly higher frequency of naïve T cells (CCR7+ CD45RA+) in LTS-NS subjects (p = 0.0066). We observed a trend towards higher levels of T_EM_ (CCR7−CD45RA−) cells in the LTS-SS group although this was not significant (p = 0.2). There was no difference in the levels of T_CM_ (CCR7+ CD45RA−) or T_EMRA_ (CCR7−CD45RA+) cells between the two groups (Figure 1A). We characterized epitope-specific CD8+ T cells for maturation profiles using intracellular cytokine staining. No differences in the maturational profiles of epitope-specific CD8+ T cells between the two groups were observed (Figure 1B).

### Striking Differences in CD4+ T cell Maturational Profiles between Progression Groups

**Figure 2:**
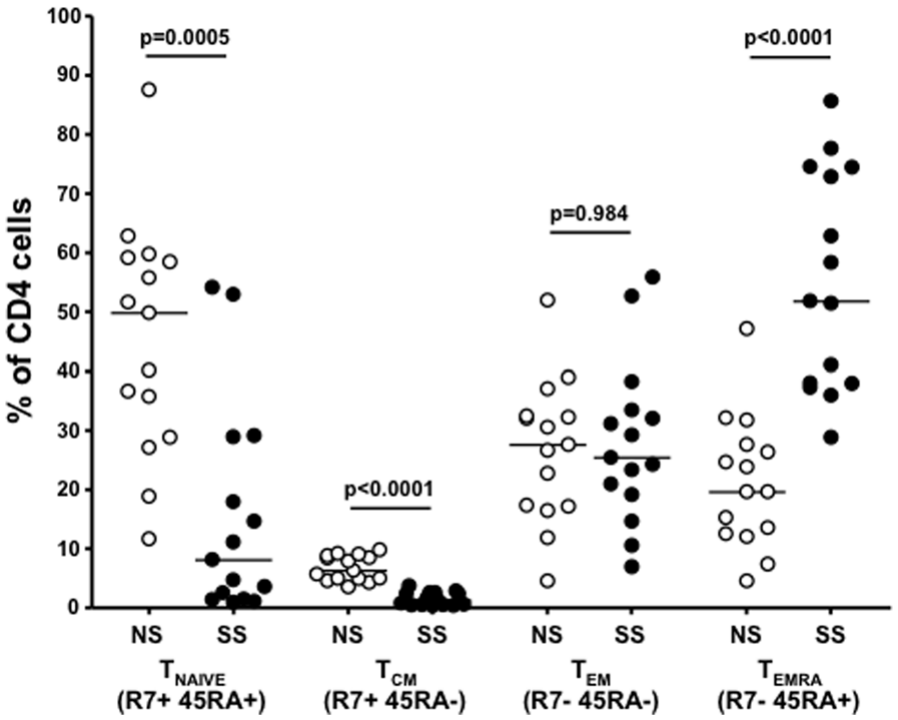
Comparison of differentiation profiles of total CD4 T cells between progression groups. We used expression of CCR7 and CD45RA to categorize CD4 T cells into one of four differentiation phenotypes. Filled circles (•) represent LTS-SS patients and empty circles (○) represent LTS-NS patients. The line in each column represents the median and the differentiation phenotype is beneath each column.

We next analyzed the characteristics of CD4+ T cells in these subjects. CD4+ cells were defined as CD3+CD8− cells. In a different panel with all three markers we verified that, on average, 93% of CD3+CD8− cells were CD4+. Remarkable differences in the maturational profiles of CD4+ T cells between the two progression groups were observed. As shown in Figure 2, subjects in the LTS-NS groups had much higher frequencies of naïve and central memory CD4+ T cells, which were highly statistically significant (p = 0.0005 and p<0.0001). There was no difference in the levels of effector memory CD4+ T cells (p = 0.984). Interestingly, subjects in the LTS-SS group had significantly higher levels of T_EMRA_ cells than LTS-NS subjects, which was also highly statistically significant (p<0.0001).

**Figure 3:**
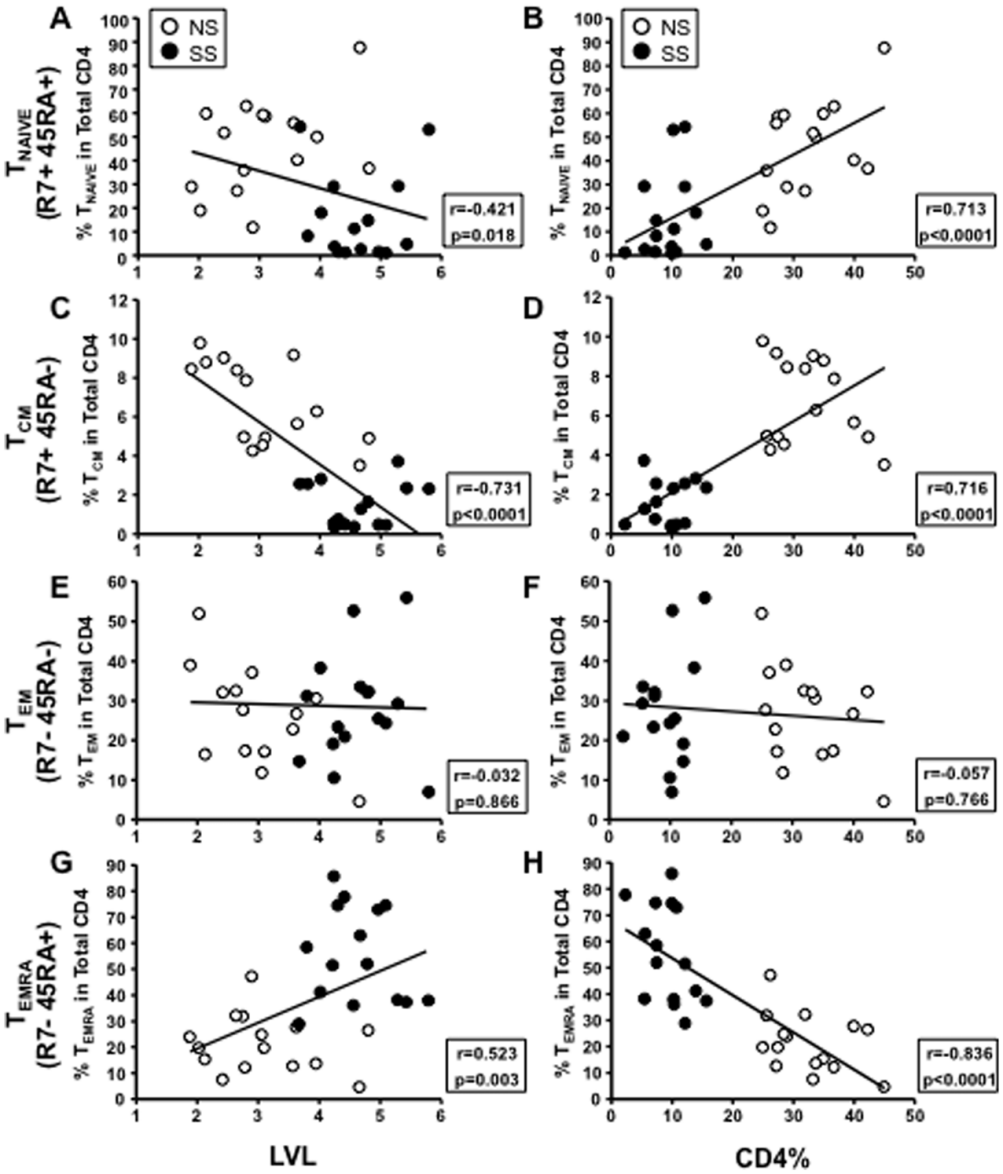
Correlations between frequency of CD4 T cell subsets and clinical characteristics. Log viral load (LVL) versus frequency of CD4 T cell subsets are shown in **A**, **C**, **E**, and **G**. CD4% (of total white blood cells) versus frequency of CD4 T cell subsets are shown in **B**, **D**, **F**, and **H**. The CD4 T cell subset analyzed is at the beginning of each row.

Additionally, the levels of T_NAIVE_ cells and T_CM_ cells showed a strong positive correlation with CD4% (Spearman r = 0.713 and 0.716, respectively, and p<0.0001 for both) and negatively with LVL (Spearman r = −0.421, p = 0.018; and Spearman r = −0.731, p<0.0001, respectively) (Figure 3A–D). In contrast, the levels of T_EMRA_ cells were strongly negatively correlated with CD4% (Spearman r = −0.836, p<0.0001) and positively correlated with LVL (Spearman r = 0.0026, p = 0.0026) (Figure 3G and H).

**Figure 4:**
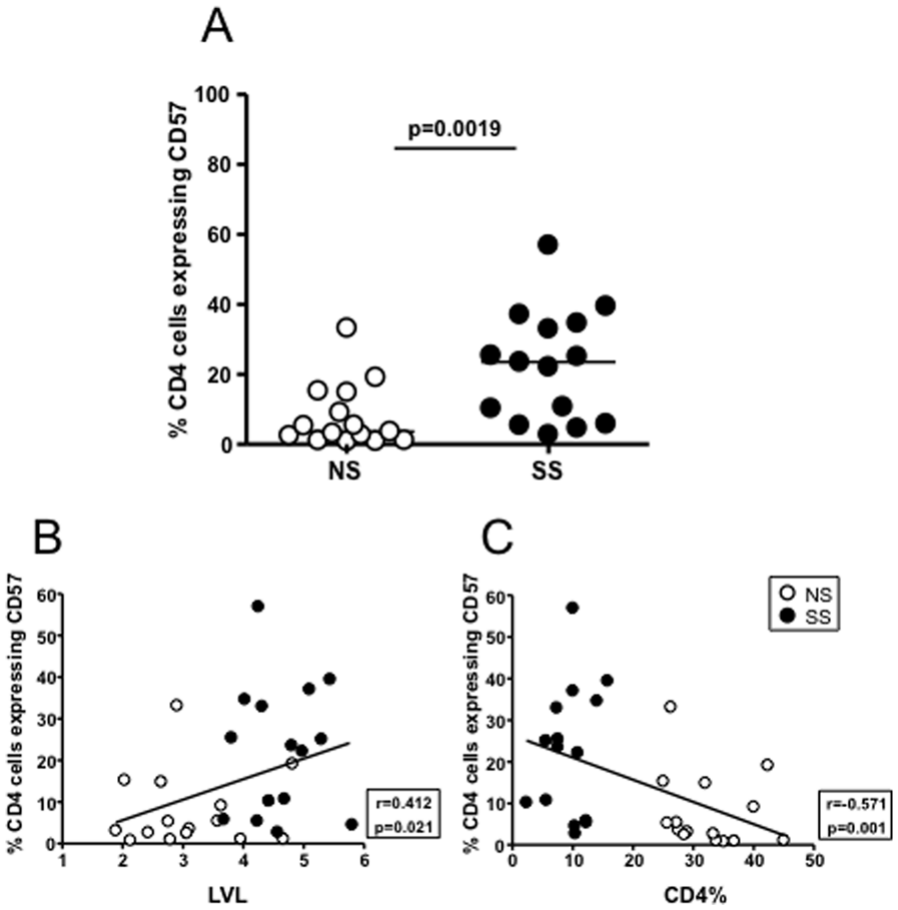
Association between CD57 expression on CD4 T cells and disease progression. **A.** Comparison of CD57 expression on CD4 T cells between progression groups. **B.** Correlation between log viral load (LVL) and CD57 expression on CD4 T cells. **C.** Correlation between CD4% and expression of CD57 on CD4 T cells.

In order to further characterize these CD4+ T cells, levels of CD57 expression were also measured. CD57 has been described a marker of T cell senescence [22]. CD57 expression on CD4+ T cells from LTS-SS subjects was much higher than that seen on CD4+ T cells from the LTS-NS group, (p = 0.0025) (Figure 4A). The frequency of CD4+ T cells expressing CD57 was also negatively correlated with CD4% (Spearman r = −0.572, p = 0.001) and positively correlated with LVL (Spearman r = 0.41, p = 0.02) (Figure 4B and C).

## Discussion

Several recent studies have suggested that qualitative characteristics of the HIV-1-specific T cell are associated with protection, viral control, and rate of disease progression [8], [20], [21], [23], [24]. To our knowledge this is the first study to compare the qualitative characteristics of total CD8+ and CD4+ as well as HIV-specific T cell responses in perinatally infected children with different levels of disease progression, with a significant finding relating specific CD4+ T cell profiles with disease progression rate. Moreover, unlike previous studies which have focused on individuals of Northern European descent, this work concentrated primarily on African Americans and Hispanics.

The children in this study, all older than 10 years of age and thus considered long-term survivors (LTS), were categorized, based on CD4+ T cell percentage levels, into those with no immune suppression (LTS-NS) and those with severe immune suppression (LTS-SS), based on previously published CDC guidelines [19]. We observed a highly significant increase in the frequency of naïve CD8+ T cells (T_NAIVE_) in the LTS-NS subjects (p = 0.0066), compared to the LTS-SS subjects, but no differences in any other CD8+ T cell subsets. The differentiation profiles of Gag-specific CD8+ T cells were similar between the progression groups.

The most striking finding of this study was the CD4+ T cell differentiation profiles between the two progression groups. Subjects in the LTS-NS group had significantly higher levels of naïve T cells (T_NAIVE_) and central memory (T_CM_) CD4+ T cells than LTS-SS children ( = 0.0005 and p<0.0001, respectively). In contrast, children in the LTS-SS group had significantly higher levels of effector memory RA+ (T_EMRA_) cells (p<0.0001). These data suggest that disease progression in these vertically infected younger patients is different than adults and is associated with a shift towards greater T_EMRA_ cell numbers.

The shift towards greater T_EMRA_ cells could represent a disproportionate loss of certain T cell subsets in LTS-SS subjects consistent with published data. A recent study showed that CD4+ cells with the T_EMRA_ phenotype (CCR7−CD45RA+) were more prevalent in HIV-1-infected individuals than in uninfected controls [25]. They described that these cells were resistant to *in vitro* infection by CCR5-tropic strains of HIV-1, despite robust expression of CCR5. Loss of Naïve T cells was also observed in a study of HIV infected infants. In this study, there was an association between rapid disease progression and decreases in naïve cells but with little effect on memory cells. The loss of naïve cells appeared to be mediated through thymic dysfunction [26], [27]. Our cohort consists of long term survivors of vertically acquired HIV-1 infection who were infected perinatally. The loss of naïve and central memory cells and increased frequency of T_EMRA_ cells could therefore be due to a combination of a greater susceptibility to HIV-1 related cell death, together with thymic dysfunction. Future studies would need to address the relative contributions of these conditions to the observations.

The greater susceptibility of naïve and central memory T cells to loss could potentially be due to effects of chronic immune stimulation from HIV-1. Additional studies would also benefit from analyses looking at cell surface activation markers such as CD38 or HLA-DR. The loss of naïve and central memory T cells, with the accumulation of T_EMRA_ cells, suggests altered T cell maturation kinetics or a change in T_EMRA_ lifespan in progressing subjects. We observed a greater frequency of CD4+CD57+ cells in the LTS-SS group that correlated negatively with CD4% and positively with viremia. CD57 is a marker of senescence. The relationship of the increased CD57+ frequency with other markers of disease progression corroborates the proposed detrimental effect of maturation. This suggests an inability of homeostatic mechanisms to maintain the appropriate proportion of T cell phenotypes necessary for HIV-1 control in progressing subjects and could be related to the accumulation of T_EMRA_ cells.

This study suggests that T cell maturation patterns are significantly different in perinatally HIV-1-infected children with different levels of disease progression, but there are some caveats to be considered. The relatively small number of subjects in the study precludes us from applying these findings to a general pediatric population. The subjects were not all on the same antiretroviral treatment regimens. However, we attempted to minimize these concerns by choosing subjects for the study who: 1) did not receive HAART in the first two years of life; 2) had some levels of ongoing viral replication; and 3) were ARV-experienced, except for two patients. Another equalizing factor is that both groups, as a whole, had generally similar treatment adherence rates. We also cannot be completely confident that the staining protocol does not potentially alter the pattern of staining of CD4+ T cells, which would be avoided by staining antigen specific cells with tetramers. However, few class II tetramers are now available for such studies.

We observed an extremely strong correlation between increased CD4+ T_EMRA_ cells and more severe immunological suppression. Although this suggests that these cells are accumulating during progressive infection, more research into this area is needed. The previously observed resistance of these terminally differentiated cells to R5 tropic strains of HIV-1 is intriguing and may be part of the explanation why these patients are long-term survivors despite persistently low CD4+ T cell levels. These findings could be of importance to the field of pediatric HIV-1 immunology as well as the larger field of HIV-1 vaccine design.

## Materials and Methods

### Ethics Statement

The research involving human participants reported in this study was approved by the University of California San Francisco (UCSF) and Albert Einstein College of Medicine (AECOM) institutional review boards IRB, with the approval number H11613–19149. Informed written consent was obtained for all subjects. All clinical investigation were conducted according to the principles expressed in the Declaration of Helsinki.

### Patient Sample Characteristics

All subjects attended the Pediatric HIV clinic at Jacobi Medical Center in the Bronx, NY. The vast majority of attendees of the Jacobi Pediatric HIV Clinic are either African-American or Hispanic. Stored samples from these patients were selected for this study based on clinical characteristics that allowed them to be classified according to previously published CDC guidelines [19], as explained below. Heparinized whole-blood samples were obtained from 58 subjects after informed consent, based on local Institutional Review Board-approved protocols. Plasma HIV-1 RNA was measured with the Amplicor HIV-1 Monitor with a lower limit of quantification at 50 copies RNA/ml (Roche Diagnostic Systems, Branchburg, NJ).

All subjects were perinatally infected and over 10 years of age, and can thus be defined as long-term survivors (LTS). Since all of the patients were born in an era prior to the availability of pediatric HAART (1996 or before), none of the subjects received potent, suppressive combination therapy during the first two years of life. All of the patients, with the exception of 2 children, were either taking antiretrovirals (ARVs) or were ARV-experienced, at the time of sampling. Additionally, all subjects were viremic at time of sampling and most had variable treatment adherence rates. There were no significant differences in treatment regimen or adherence levels between the two clinical groups.

### Clinical Categorization

The general consensus in the pediatric HIV field is that the CD4% value is the most valuable marker of disease progression and is used by the CDC to classify levels of disease status in HIV infected children [19]. In this study, patients were categorized into two groups based on CD4% values, according to CDC guidelines [19]. Subjects with a sustained CD4%≥25% were considered LTS with No Evidence of Immune Suppression (LTS-NS). Those with a sustained CD4%≤15% were considered LTS with Severe Immune Suppression (LTS-SS).

Patient histories were used to categorizing patients. Only patients that had always possessed CD4% levels very near or above 25% were considered as an LTS-NS subject. Patients that possessed CD4% levels very near or below 15% for several clinic visits were grouped as an LTS-SS subject. A window was delineated for each patient within which samples were assayed immunologically. We chose a period of time in which samples were available and the patient was as clinically stable as possible, as defined by CD4% and viral loads. We acknowledge that a range of immunosuppresion exists within each category, but we have given an overall classification of “no evidence” and “severe” to help distinguish the groups.

### Multi-parameter flow cytometry

Single peptides, comprising the HXB2 sequence of Gag (AIDS Research and Reference Reagent Program, NIH), previously identified as containing targeted epitopes were used as the antigenic stimulus (Sharp et al., in preparation). The negative control was media alone and the positive control was Staphylococcal Enterotoxin B (SEB). We also stimulated cultures with a peptide pool consisting of well-identified HLA class I restricted CMV, EBV and Influenza epitopes (CEF) in patients that had previously demonstrated reactivity.

Briefly, cryopreserved PBMC were thawed and cells were stimulated for one hour with either: media alone, antigen, or positive control. The antibody, CD107 α/β-PECy5 was added with stimulation. Brefeldin A (Sigma-Aldrich, St. Louis, MI, USA) was then added at a concentration of 5 µg/ml and cells incubated overnight. The next day, PBMC were washed and stained with antibodies, in combination, against CD4-Alexa700, CD8-Pacific Blue, CD45RA-biotin, CCR7-PECy7, CD57-PECy5, and a live/dead marker emitting in the aqua wavelength for 20 minutes at 4°C. Cells were washed twice and stained with the secondary antibody streptavidin-Qdot655 for 20 minutes at 4°C. Cells were then washed and fixed in 2% paraformaldehyde. The cells were permeabilized using FACS Perm solution (BD Biosciences), washed and stained using antibodies, in combination, against CD3-ECD and IFN-γ-APC for 30 minutes at 25°C. Following staining, the cells were washed, fixed in 1% paraformaldehyde, and collected on a BD LSR-II using FACS DIVA software (BD Biosciences). Data was analyzed using FlowJo (TreeStar).

### Gating Strategy

In all analyses a forward scatter (FSC)-height versus FSC-area plot to exclude all cell conjugates was used. Dead cells were then excluded by only gating on cells negative for the live/dead marker. A FSC-area vs. side scatter (SSC)-area plot was used to define the lymphocyte gate. T cells were selected by gating on CD3+ lymphocytes, followed by selection of CD8+ cells by gating on CD3+CD8+ cells. CD4+ cells were defined as CD3+CD8− cells. In panels that contained CD3, CD4, and CD8 antibodies, we verified that, on average, 93% of CD3+CD8− cells were CD4+. IFN-γ+ cells were defined using an APC “fluorescence minus one” (FMO) sample. Quadrant gates were set for expression of CCR7 and CD45RA by using a QDot655 FMO and a PECy7 FMO. IFN-γ+ cells were further analyzed for expression of T-cell memory markers in a CCR7 versus CD45RA.

### Statistical Methods

A median (interquartile range) was used as a measure of central tendency for continuous variables. We employed the Mann-Whitney two-tailed t-test for all simple comparisons between two groups. The Spearman Rank correlation test and linear regression analyses were used to explore associations between 2 continuous variables. Differences between categorical data were calculated using Fisher's exact test. We considered a p-value of <0.05 significant. All statistical analyses were performed using the GraphPad Prism 4.03 software package (La Jolla, CA).
